# Immunomodulatory effects of selenium‐enriched peptides from soybean in cyclophosphamide‐induced immunosuppressed mice

**DOI:** 10.1002/fsn3.2594

**Published:** 2021-09-21

**Authors:** Jian Zhang, Siwei Gao, He Li, Mengdi Cao, Wenhui Li, Xinqi Liu

**Affiliations:** ^1^ National Soybean Processing Industry Technology Innovation Center Beijing Advanced Innovation Center for Food Nutrition and Human Health Beijing Engineering and Technology Research Center of Food Additives Beijing Technology and Business University Beijing China; ^2^ Chinese Academy of Inspection and Quarantine Beijing China

**Keywords:** cyclophosphamide, immunomodulatory, immunosuppressed mice, selenium‐enriched soybean peptides

## Abstract

In this study, selenium‐enriched soybean peptides (<3 kDa, named Se‐SPep) was isolated and purified from the selenium‐enriched soybean protein (Se‐SPro) hydrolysate by ultrafiltration. The in‐vivo immunomodulatory effects of Se‐SPep were investigated in cyclophosphamide‐induced immunosuppressed mice. Se‐SPep treatment could alleviate the atrophy of immune organs and weight loss observed in immunosuppressive mice. Besides, Se‐SPep administration could dramatically improve total protein, albumin, white blood cell, immunoglobulin (Ig) M, IgG, and IgA levels in blood. Moreover, Se‐SPep strongly stimulated interleukin‐2 (IL‐2), interferon‐gamma (IFN‐γ), nitric oxide (NO), and cyclic guanosine monophosphate productions by up‐regulating mRNA expressions of IL‐2, IFN‐γ, and inducible NO synthase in spleen tissue. Furthermore, Se‐SPep exhibits more effective immunomodulatory activity compared to Se‐SPro and SPep. In conclusion, Se‐SPep could effectively enhance the immune capacity of immunosuppressive mice. These findings confirm Se‐SPep is an effective immunomodulator with potential application in functional foods or dietary supplements.

## Graphical abstract

Low‐molecular weight peptides Se‐SPep were prepared from selenium‐enriched soybean. Se‐SPep has immunomodulatory effects on immunosuppressed mice. Supplementation of Se‐SPep possesses therapeutic potential for immunosuppression, and may potentially act as a promising immunomodulator.

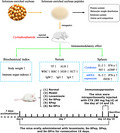

## 1 INTRODUCTION

The immune system of the human body essentially prevents and controls infection and neoplasia by cellular and humoral mechanisms (Parkin & Cohen, 2001). Cellular immunity mainly involves T lymphocytes and B lymphocytes, while humoral immunity plays a major role with specific antibodies produced by B lymphocytes after receiving antigen presentation. The immune functions may be adversely affected by many factors, including malnutrition, oxidative stress, exogenous pathogens, and antigens (De la Fuente, 2002; Li et al., 2019). In recent years, research on enhancing human immunity by ingesting food has attracted increasing attention worldwide. Therefore, the development of functional foods or nutritional supplements is an important and practical approach for regulating immune functionality.

Swedish chemist Jöns Jacob Berzelius discovered selenium (Se) in 1817 (Lu & Holmgren, 2009). Since 1957, Se has been considered an essential trace element for many life forms and is crucial for human health. It has been proven that Se, as an essential micronutrient with antioxidant properties, can improve immune functions in the body by regulating humoral immunity, cellular immunity, and nonspecific immunity (Carlson et al., 2010; Esmail et al., 2020). Se plays an essential physiological role in the body in the form of selenoprotein (Zhang et al., 2021). When dietary Se intake is insufficient, the selenoprotein content in the body tissues is significantly reduced, inhibiting the growth and development of immune cells. This leads to a decrease in the immune capacity of the body, causing oxidative stress in tissue cells and even inducing cell apoptosis. An insufficient intake of Se has been shown to be related to immune dysfunction in animals (Qin et al., 2020; Zhang, Liu, et al., 2020). Studies on selenoprotein S explained Se deficiency and poor plasma Se levels can cause cardiovascular diseases by decreasing selenoprotein levels (Chi et al., 2021). A growing number of studies have confirmed that Se strengthens immunity, increases antibody levels, and improves nonspecific immunity (Arvilommi et al., 1983). Se can also significantly improve the survival and phagocytosis rates of phagocytic cells, enhancing cellular immune functionality (Ndiweni & Finch, 1996). A research team from the University of Surrey in the United Kingdom introduced the relationship between the coronavirus disease 2019 (COVID‐19) cure rate (reported in 17 cities) and the distribution of Se in China. The study revealed that the COVID‐19 cure rates tended to be higher in areas with abundant Se content (Zhang, Taylor, et al., 2020). Supplementing Se‐rich food is also excellent for fighting COVID‐19 (Bermano et al., 2021; Seale et al., 2020). Food is the primary source for human Se ingestion. However, Se is not evenly distributed globally, and its consumption in low‐Se areas is highly insufficient. Due to improvements in bioavailability, immunoregulatory activity, antioxidant capacity, and lower toxicity, the organic forms of Se can better meet dietary requirements than the inorganic forms (Tinggi, 2003). Improving the absorption and utilization of organic Se in the body, physiological functionality is attracting considerable research focus. In recent years, the use of functional foods or adjuvants derived from natural dietary sources as an alternative to chemical or biosynthetic drugs for the prevention and treatment of diseases has received increasing attention in an attempt to avoid the toxicity and side effects associated with conventional treatments (Kim & Wijesekara, 2010; Yu et al., 2019).

Soybeans exhibit a certain ability to accumulate Se. It can actively absorb Se in the environment while converting inorganic Se into organic Se (Mateos‐Aparicio et al., 2008). Furthermore, Se can replace the sulfur element in sulfur‐containing amino acids, nonspecifically combining with them to form seleno amino acids, which are present in selenoproteins, facilitating various physiological activities (Bellinger et al., 2009). Additionally, 80% of the Se enriched in soybeans is combined with protein, while as much as 82% of Se is mainly in the form of seleno amino acids (Sathe et al., 1992). Soybean peptide (SPep) is a mixture obtained by separating and purifying soybean protein (SPro) after enzymolysis by protease. It can enhance the immune regulation ability of the body by adjusting the immunoglobulin content and immune factors (Zhang, Fu, et al., 2020). Moreover, the absorption and transport speed of SPep in the body are higher than those of proteins and free amino acids containing the same number of amino acids (Dei Piu et al., 2014). A previous study gavaged *SD* rats with Se‐enriched soybean peptide (Se‐SPep) and Se‐enriched soybean protein (Se‐SPro). The results revealed a faster absorption rate of Se‐SPep in the body, providing a scientific basis for nutritional Se enhancement (Gao et al., 2021). Therefore, compared with inorganic Se, Se‐SPep displays a faster absorption rate, and substantial functional activity in the body, while being safe and nontoxic, providing an efficient Se‐enriched dietary supplement.

This article investigates the preventive immune effect of Se‐SPep by examining the bodyweight, organ index, peripheral blood routine, serum immunoglobulin content, spleen immune factor content, and gene expression of cyclophosphamide (CTX)‐induced immunosuppressed mice. It also explores the difference between the effect of Se‐SPep and Se‐SPro, providing a theoretical scientific basis for the development and utilization of Se‐SPep as dietary immune supplements.

## 2 MATERIALS AND METHODS

### 2.1 Materials and chemicals

Se‐enriched soybean was purchased from Enshi Se‐Run Health Tech Development Co., Ltd. CTX was obtained from Shanghai Yuanye Bio‐Technology Co., Ltd. Levamisole was purchased from Novozymes (China) Investment Co., Ltd. The enzyme‐linked immunosorbent assay (ELISA) kits for the total protein (TP), albumin (ALB), immunoglobulin M, G, and A (IgM, IgG, and IgA), interleukin‐2 (IL‐2), and interferon‐γ (IFN‐γ) were purchased from Beijing Solarbio Science & Technology Co., Ltd. The assay kit for nitric oxide (NO) and cyclic guanosine monophosphate (cGMP) were purchased from Sigma‐Aldrich. All chemical reagents were of analytical grade.

### 2.2 Preparation of the Se‐SPep

The Se‐SPep was prepared as described in a previous report (Gao et al., 2021). Briefly, the Se‐enriched soybeans were ground, defatted, and dried to obtain the soybean kernel flour, which was then mixed with deionized water at a ratio of 1:20 (w/v), the pH was adjusted to 8.0 by adding 2 N NaOH. The slurry was stirred at 40°C for 2 h, after that it was centrifuged at 2100 *g* for 20 min. The supernatant was adjusted to pH 4.5 by adding 2 N HCl and stored for 30 min at 4°C. After centrifugation at 2100 *g* for 20 min, the Se‐SPro product was obtained. The Se‐SPro was added to water until reaching a protein content of 8%, followed by digestion with alkaline protease, neutral protease, and papain at a ratio of 2:1:1. The proteases were added at 0.2% of the Se‐SPro weight, after that hydrolysis was performed at 50°C for 4 h. The digest was heated at 95°C for 15 min to deactivate the enzyme. The degree of hydrolysis in optimal conditions was 68.53%. The hydrolysate was then centrifuged at 1700 *g* for 15 min, and the supernatant was filtered through a 0.45 μm microporous membrane.

The molecular weight distribution of Se‐SPep was determined following a previously reported method (Zhang, Li, et al., 2020). The standard molecular weight samples containing aprotinin (6500 Da), bacitracin (1422 Da), and Gly–Gly–Tyr–Arg (451 Da) were passed through 0.22 μm filters and successively loaded into a Superdex 200 10/300 GL column. The chromatographic analysis was performed using an ÄKTA pure system (AKTA pure 25, Cytiva). The PBS (0.05 M and pH 7) mobile phase was eluted at a flow rate of 0.5 ml/min and detected at a wavelength of 220 nm.

The amino acid compositions of the Se‐SPro and Se‐SPep were determined by using an amino acid analyzer (Biochrom 30+ amino acid analyzer; BioChrom Ltd) with a Na cation exchange column (8 mm, 4.6 × 200 mm), which was purchased from Waters Corporation. The amino acids were derivatized with ninhydrin reagent after they passed through the exchange column. The absorbance of the resulting material was measured at 440 nm (for proline [Pro]) and 570 nm (for all other amino acids).

Finally, the supernatant was fractionated using a molecular weight cut‐off of 3 kDa (PLCC, Millipore). The <3 kDa peptide fractions were collected and lyophilized for further study. The preparation method of SPep was the same as above.

### 2.3 Animals and diets

A total of 60 male BALB/c mice (6 weeks old), weighing 20 ± 2 g, were procured from SPF (Beijing) Biotechnology Co., Ltd. (laboratory animal production license number: SCXK [Jing] 2016‐0002). All mice are housed at constant temperature (24 ± 0.5°C) and humidity (50%–60%), with a 12‐h/12‐h day/night interval. The animals were fed a standard laboratory pellet diet and had free access to water. The mice were used in the experiments after a 1‐week acclimation period. The experimental protocol was approved by the Institutional Animal Care and Use Committee at the Pony Testing International Group Co., Ltd. (PONY‐2021‐FL‐02).

### 2.4 Experimental procedures

**FIGURE 1:**
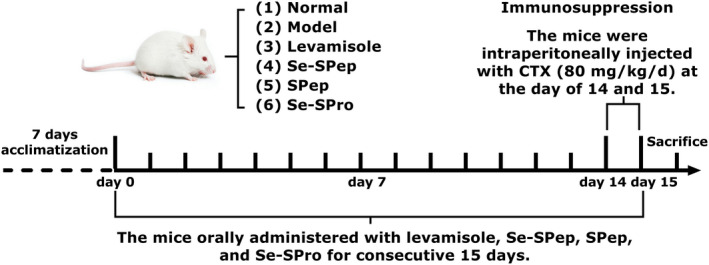
Experimental animal protocol used in this study

The experimental animal protocol is shown in Figure 1. After 1‐week acclimatization, the mice were randomly divided into six treatment groups (*n* = 10 per group): (1) Normal, (2) Model, (3) Levamisole (an immunopotentiating agent used as a positive drug), (4) Se‐SPep, (5) SPep, and (6) Se‐SPro. The bodyweights of the mice were measured once every 3 days. The animals in groups (4), (5), and (6) were orally administered with Se‐SPep, SPep, and Se‐SPro, respectively, for 15 days. Furthermore, groups (1) and (2) received the same volume of distilled water via oral gavage, while group (3) received 10 mg/kg bodyweight levamisole via oral gavage. The Se‐SPep and Se‐SPro doses were 377 and 474 mg/kg once a day, which was equivalent to the required 3.57 μg/kg Se per day for adults (Kieliszek & Blazejak, 2016). The SPep dose was equivalent to the protein content of Se‐SPep. The immunosuppression mouse model induced by CTX was established according to the previous method (Xu et al., 2016). CTX (80 mg/kg/days) was intraperitoneally injected into the mice in groups (2) to (6) on days 14 and 15. Group (1) was administered intraperitoneally with the same volume of physiological saline. After the last injection, the mice were starved for 24 h but given free access to water. The mice were weighed and sacrificed by cervical dislocation. Thymus and spleen tissues were immediately dissected, washed in precooled normal saline at 4°C, dried using filter paper, and weighed.

### 2.5 Determination of the bodyweight and immune organ index

From the day of allocation to experimental groups, the bodyweights of the mice were recorded once every 3 days until the end of the experiment. On the 16th day after different treatments, the BALB/c mice in each group were sacrificed via cervical dislocation. The spleens and thymuses were removed and weighed. The organ index was calculated as follows:

Organ index (mg/g) = Organ weight (mg)/Bodyweight (g).

### 2.6 Plasma protein measurements

Blood was collected from the eyes of the mice and left to stand for 30 min, after that it was centrifuged at 4°C at 1000 *g* for 10 min. After the serum was collected, the TP and ALB concentrations were analyzed using an automatic biochemical analyzer (BS‐350E, Shenzhen Mindray Bio‐medical Electronics Co., Ltd.).

### 2.7 Hematological analyses

Blood was collected for the eyes of the mice 24 h after the last drug administration. The white blood cells (WBC), red blood cells (RBC), hemoglobin concentration (HGB), hematocrit (HCT), mean corpuscular volume (MCV), mean corpuscular hemoglobin (MCH), mean corpuscular hemoglobin concentration (MCHC), and platelet number (PLT) were determined within 24 h using a hematology analyzer (HEMAVET 950, Drew Scientific Group).

### 2.8 Assay of immunoglobulins in the serum

After centrifugation, serum was obtained from the blood collected from the eyes. The serum immunoglobulin (IgM, IgG, and IgA) concentrations were detected using ELISA kits according to the instructions of the manufacturer. The optical density (OD) value of the solution was measured at 450 nm using an automatic microplate reader (Infinite 200 Pro Nanoquant, Tecan).

### 2.9 Determination of the cytokine levels in the spleen

The spleens of the BALB/c mice were removed in a sterile environment and washed twice with phosphate buffer saline (PBS). The spleen tissue was accurately weighed, homogenized, and then placed in a tube with 2 ml saline. Supernatants of the spleen were collected via centrifugation at 1800 *g* at 4°C for 15 min. The IL‐2, IFN‐γ, NO, and cGMP content in the spleen were determined using an ELISA kit according to the instructions.

### 2.10 Quantitative real‐time polymerase chain reaction analysis

The mRNA expression levels of IL‐2, IFN‐γ, and inducible NO synthase (iNOS) were determined using quantitative real‐time polymerase chain reaction (qRT‐PCR). The spleens of the BALB/c mice were removed in a sterile environment and washed twice with PBS buffer. The total RNA was extracted from spleens using the Trizol reagent. A microplate spectrophotometer system (Nanodrop 2000c, Thermo Fisher Scientific) was used to determine the RNA concentration and purity. The total RNA (2 μg) was converted into cDNA using PrimeScript RT Master Mix (Takara‐bio), while qRT‐PCR amplification was performed using Probe Qpcr Mix (Takara‐bio). The β‐actin gene was used for the normalization of IL‐2, IFN‐γ, and iNOS mRNA expression. The relative expression levels of the target genes were calculated based on 2^−ΔΔCt^ according to the specifications of the manufacturer (Ren et al., 2017).

### 2.11 Statistical analysis

Data were expressed as the mean ± standard deviation (*SD*). The results were analyzed with one‐way analysis of variance (ANOVA) followed by Tukey's method using SPSS 23 software, while graphs were created using Graph Pad Prism 7 software.

## 3 RESULTS

### 3.1 Characterization of Se‐SPep

**FIGURE 2:**
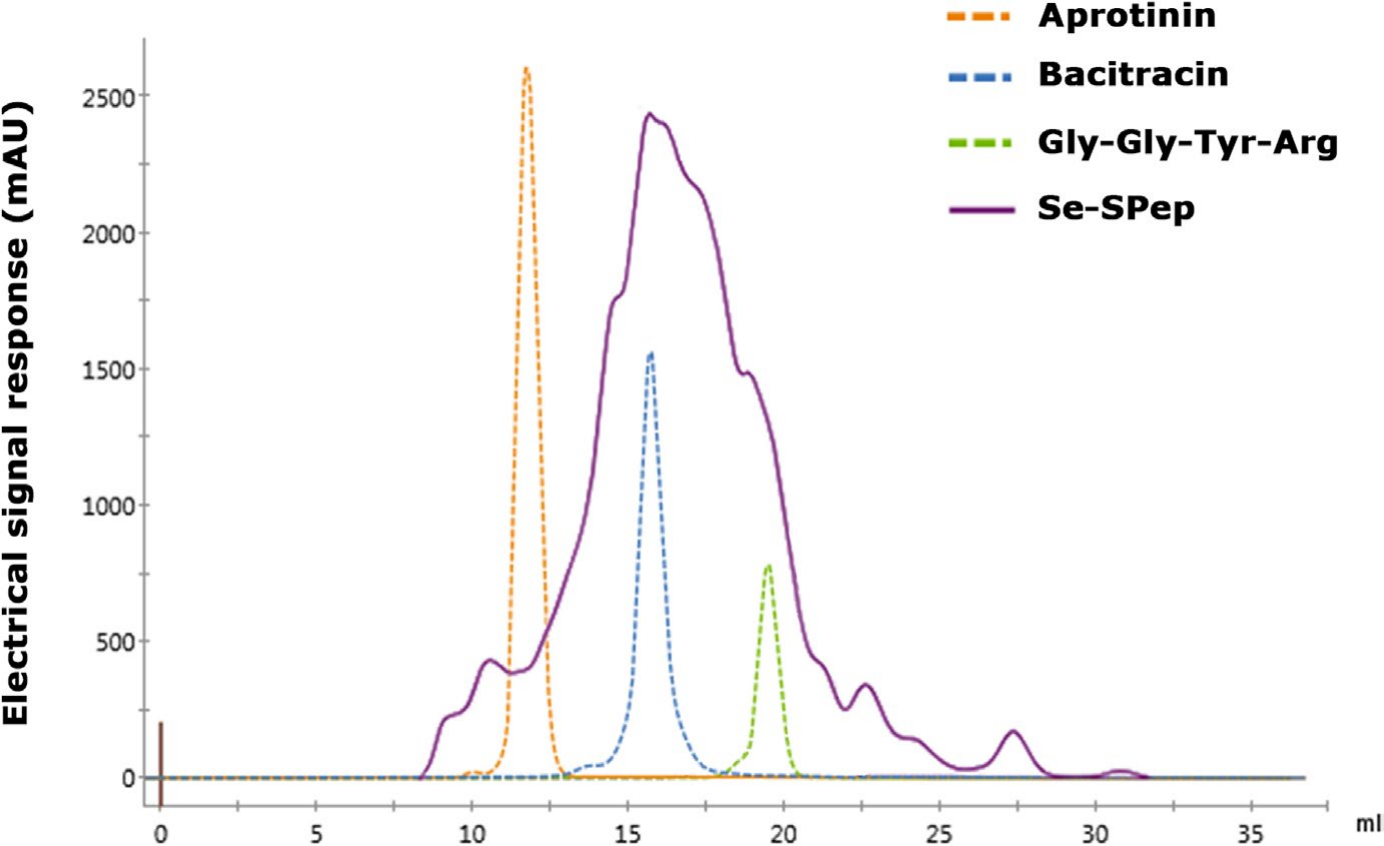
Sephadex G‐25 chromatograms of the standard molecular weight samples and Se‐SPep. The standard molecular weight samples: Aprotinin‐6500 Da, Bacitracin‐1422 Da, and Gly–Gly–Tyr–Arg–451 Da

The protein content of Se‐SPro and Se‐SPep was determined using the Kjeldahl method (Kjeltec 8000, FOSS Analytical A/S) and were 89.81% and 85.10%, respectively. The molecular weight of peptides affects their physiological and functional properties (Yoshikawa et al., 2000). Furthermore, bioactive peptides generally correspond to protein hydrolysates and low molecular weight peptide mixtures. The ÄKTA pure system was operated by gel filtration chromatography to determine the molecular weight distribution of Se‐SPep. As shown in Figure 2, the molecular weight ratio, <3000 Da, was 88.1%. According to the GB 5009.93‐2010 national standard, the total Se content in Se‐SPro and Se‐SPep were tested using Hydride Generation‐Atomic Fluorescence Spectrometry (LC‐AFS6500, Beijing Haiguang Instrument Co., Ltd.). The Se levels in Se‐SPro and Se‐SPep were 68.5 and 110.4 mg/kg, respectively. The amino acid composition in the Se‐SPro and Se‐SPep used in the experiment were both assayed using an amino acid analyzer (Biochrom 30+, BioChrom Ltd.). From Table 1, the essential amino acids (Thr, Val, Met, Ile, Leu, Phe, His, and Lys) were accounted for 37.92% of the total amino acids in Se‐SPep. In plants, Se becomes converted to organic forms including the amino acids selenomethionine and selenocysteine (Vanda Papp et al., 2007). The methionine content of Se‐SPro and Se‐SPep was 104.42 and 189.32 μmol/g, respectively, while the cysteine content was 30.56 and 47.09 μmol/g.

**TABLE 1:** The amino acid compositions (%) in Se‐SPro and Se‐SPep

Amino acid	Se‐SPro (%)	Se‐SPep (%)
Aspartic acid^a^	11.81 ± 0.21	10.65 ± 0.08
Threonine^b^	3.83 ± 0.12	4.35 ± 0.12
Serine^a^	5.44 ± 0.18	6.41 ± 0.10
Glutamic acid^a^	17.76 ± 0.69	19.08 ± 0.21
Proline^a^	5.04 ± 0.21	3.08 ± 0.19
Glycine^a^	7.17 ± 0.21	7.47 ± 0.12
Alanine^a^	6.26 ± 0.27	7.78 ± 0.14
Cysteine ^a^	0.39 ± 0.09	0.31 ± 0.06
Valine^b^	6.26 ± 0.14	6.37 ± 0.18
Methionine^b^	1.35 ± 0.04	1.23 ± 0.11
Isoleucine^b^	5.15 ± 0.22	4.93 ± 0.18
Leucine^b^	8.17 ± 0.26	9.04 ± 0.25
Tyrosine^a^	3.13 ± 0.25	3.36 ± 0.08
Phenylalanine^b^	4.33 ± 0.10	4.86 ± 0.13
Histidine^b^	2.42 ± 0.17	2.16 ± 0.14
Lysine^b^	5.65 ± 0.23	4.97 ± 0.10
Arginine^a^	5.85 ± 0.08	3.94 ± 0.16

Note

Values are expressed as means ± *SD* (*n* = 3).

^a^ Nonessential amino acids.

^b^ Essential amino acids.

### 3.2 The effect of Se‐SPep on bodyweight and immune organ index

**FIGURE 3:**
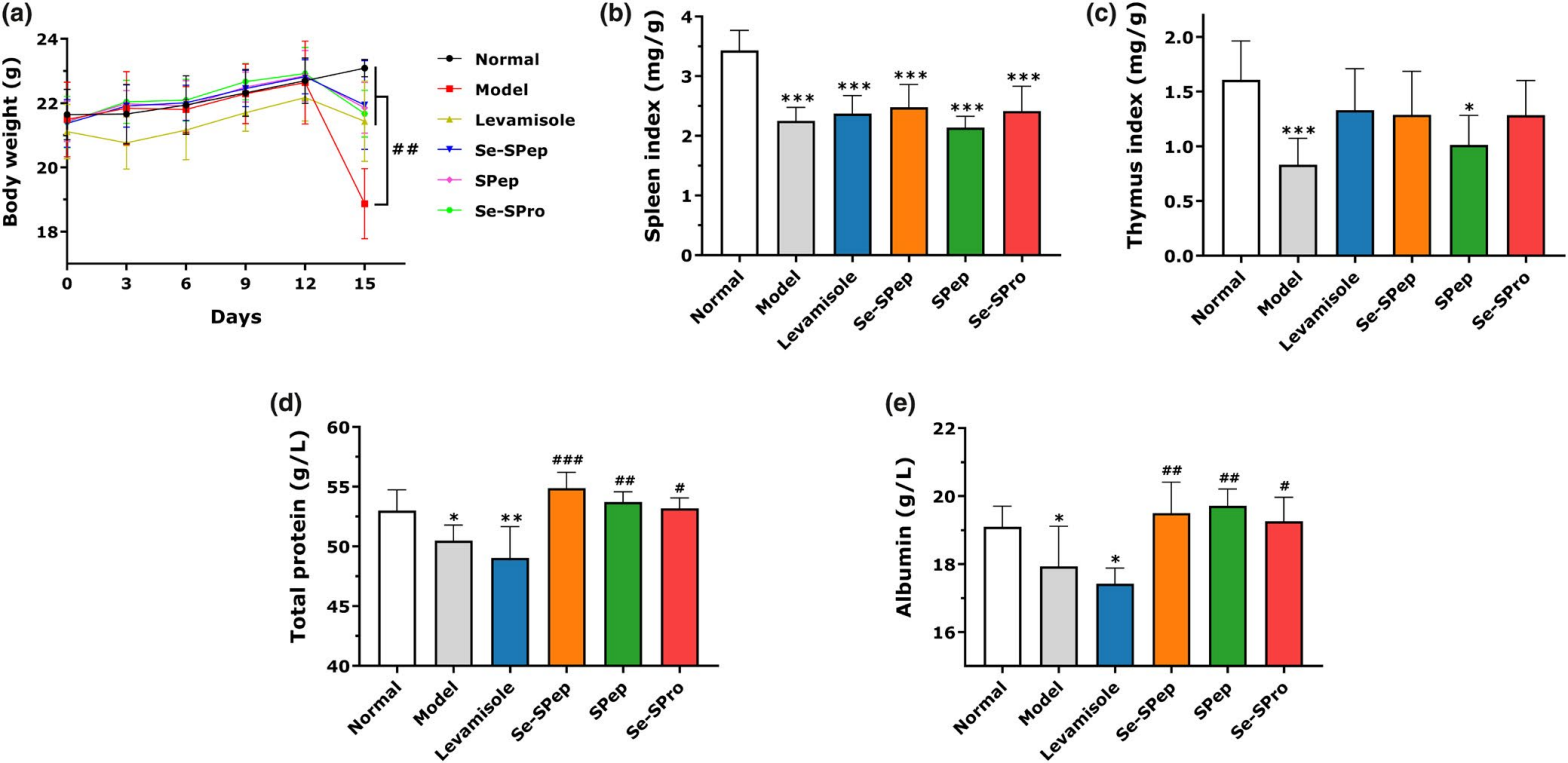
Effect of Se‐SPep on bodyweight (a), spleen index (b), thymus index (c), total protein contents (d), and albumin contents (e) in cyclophosphamide‐treated mice (*n* = 10). Compared with the Normal group: ∗*p* < .05, ∗∗*p* < .01, and ∗∗∗*p* < .001; compared with the Model group: ^#^ *p* < .05, ^##^ *p* < .01, and ^###^ *p* < .001

Cyclophosphamide is a clinical chemotherapeutic drug that exhibits an immunosuppressive effect, such as bodyweight loss and decreasing immune organ indexes in animal models (Huang et al., 2020). As shown in Figure 3a, no significant changes were evident in the bodyweight of the different groups throughout the 2‐week feeding period. The mice exhibited significant bodyweight loss after injection with CTX (*p* < .05), except for the mice in the Normal group, who gained weight. These phenomena indicated that immunosuppression was successfully induced in the mice. The bodyweight of the mice in the Levamisole, Se‐SPep, SPep, and Se‐SPro groups was significantly higher than in the Model group (*p* < .05). Therefore, the results confirmed a preventive effect on weight loss in the CTX‐induced immunosuppressed mice.

The spleen and thymus are pivotal components of the immune system. As shown in Figure 3, the spleen (Figure 3b) and thymus (Figure 3c) indexes of the Model group decreased significantly compared with the Normal group (*p* < .05). These findings indicated that CTX caused atrophy in the immune organs while confirming the validity of the established immunosuppressive model. The spleen index of the Levamisole, Se‐SPep, and Se‐SPro groups was higher than that of the Model group, but there was no significant difference. Li et al. (2018) indicated the remission of CTX‐induced spleen atrophy by low dose Se‐enriched *Grifola frondosa* is not obvious. The thymus index of the Levamisole, Se‐SPep, and Se‐SPro groups showed clear improvement compared with the Model group. These results showed that Se‐SPep and Se‐SPro could alleviate the immune organ atrophy caused by CTX to a certain extent. Additionally, the spleen and thymus indexes of the Se‐SPep and Se‐SPro groups were higher than in the SPep group, with the indexes being close to those of the Levamisole group. These findings suggest that Se plays a crucial role in preventing the atrophy of immune organs.

### 3.3 The effect of Se‐SPep on plasma protein

As shown in Figure 3, the TP (Figure 3d) and ALB (Figure 3e) concentration in the serum tended to be significantly lower in the Model and Levamisole groups than in the Normal group. This indicated that CTX could significantly reduce the TP and ALB (*p* < .05) concentrations. Treatment of Se‐SPep, SPep, and Se‐SPro, significantly enhanced the TP and ALB levels in the mice serum, approaching that of the Normal group (*p* < .05). The results indicated that the mice exhibited a better nutritional status following the administration of nutritional protein supplementation.

### 3.4 The effect of Se‐SPep on hematological parameters

As shown in Table 2, after intraperitoneal injection of CTX, significantly lower values for WBC, RBC, HGB, and HCT were noted in the Model group, compared with the Normal group (*p* < .05) indicating that the immunity of the CTX‐induced mice was suppressed. Compared with the Model group, Levamisole, Se‐SPep, and Se‐SPro significantly increased WBC (*p* < .05). The WBC of the SPep group was significantly lower than in the Se‐SPep group (*p* < .05). The RBC, HGB, and HCT of mice treated with Levamisole, Se‐SPep, SPep, and Se‐SPro were markedly elevated compared to the Model group (*p* < .05). The administration of Se‐SPep and Se‐SPro restored the routine blood indexes, suggesting that these compounds protected against CTX‐induced immunosuppression.

**TABLE 2:** The effect of Se‐SPep on the hematological parameters in cyclophosphamide‐treated mice

Group	Normal	Model	Levamisole	Se‐SPep	SPep	Se‐SPro
WBC (K/μl)	4.25 ± 0.61^a^	0.98 ± 0.33^b^	1.87 ± 0.09^c^	2.21 ± 0.18^c^	1.27 ± 0.19^b^	1.74 ± 0.17^c^
RBC (M/μl)	12.05 ± 0.56^a^	7.92 ± 2.32^b^	11.39 ± 0.55^a^	11.27 ± 0.92^a^	11.14 ± 0.58^a^	11.87 ± 0.51^a^
HGB (g/dl)	18.24 ± 0.80^a^	15.10 ± 1.75^b^	17.28 ± 0.80^a^	17.77 ± 0.06^a^	17.19 ± 0.93^a^	18.30 ± 0.08^a^
HCT (%)	59.98 ± 3.53^a^	43.62 ± 11.35^b^	57.53 ± 2.62^a^	58.73 ± 3.63^a^	56.04 ± 2.28^a^	58.66 ± 3.25^a^
MCV (fl)	50.94 ± 2.04^ns^	49.72 ± 1.66	50.03 ± 1.31	48.99 ± 1.43	48.83 ± 0.69	49.29 ± 3.26
MCH (pg)	15.04 ± 0.59^ns^	15.46 ± 0.30	15.13 ± 0.32	15.51 ± 0.55	15.20 ± 0.30	15.36 ± 0.30
MCHC (g/dl)	30.49 ± 1.05^ns^	31.07 ± 1.25	30.62 ± 1.24	31.54 ± 0.65	31.21 ± 0.66	31.49 ± 1.53
PLT (K/μl)	733.80 ± 60.03^ns^	651.30 ± 23.11	802.80 ± 46.40	710.30 ± 156.50	675.30 ± 20.50	787.00 ± 25.24

Note

Values are expressed as means ± *SD* (*n* = 10).

Different superscript letters indicate significant (*p* < .05) differences among the groups. The ns indicate no significant differences among the groups.

Abbreviations: HCT, hematocrit; HGB, hemoglobin concentration; MCH, mean corpuscular hemoglobin; MCHC, mean corpuscular hemoglobin concentration; MCV, mean corpuscular volume; PLT, platelet countRBC, red blood cell count; WBC, white blood cell count.

### 3.5 The effects of Se‐SPep on the immunoglobulin levels

**FIGURE 4:**
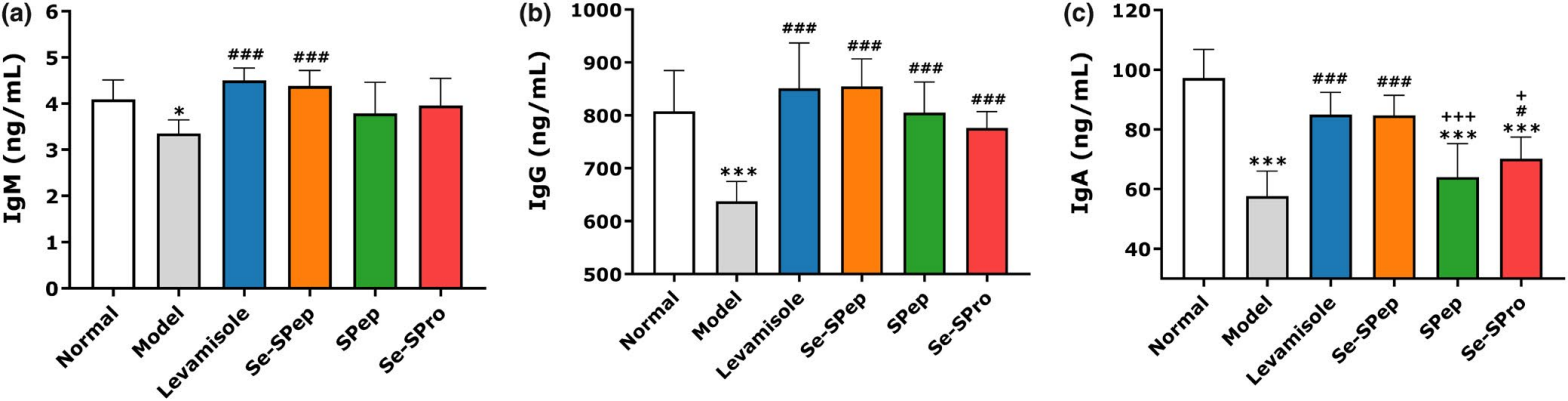
Effect of Se‐SPep on the IgM (a), IgG (b), and IgA (c) contents in the serum of cyclophosphamide‐treated mice (*n* = 10). Compared with the Normal group: ∗*p* < .05 and ∗∗∗*p* < .001; compared with the Model group: ^#^ *p* < .05 and ^###^ *p* < .001; compared with the Se‐SPep group: ^+^ *p* < .05 and ^+++^ *p* < .001

The serum IgM, IgG, and IgA levels of each group were identified to determine the effect of Se‐SPep on the humoral immunity of CTX‐treated mice. Compared with the Normal group, significantly lower IgM (*p* < .05), IgG (*p* < .001), and IgA (*p* < .001) serum levels were detected in the Model group (Figure 4). Treatment with Levamisole and Se‐SPep significantly enhanced the serum IgM, IgG, and IgA levels of the mice, approaching the values of the Normal group. Furthermore, the IgM, IgG, and IgA levels were higher in the Se‐SPep‐treated mice than in the SPep and Se‐SPro groups, while the IgA level exhibited a marked increase. These results indicated that Se‐SPep could restore serum IgM, IgG, and IgA levels in immunosuppressed mice.

### 3.6 The effect of Se‐SPep on the cytokine, NO, and cGMP levels

**FIGURE 5:**
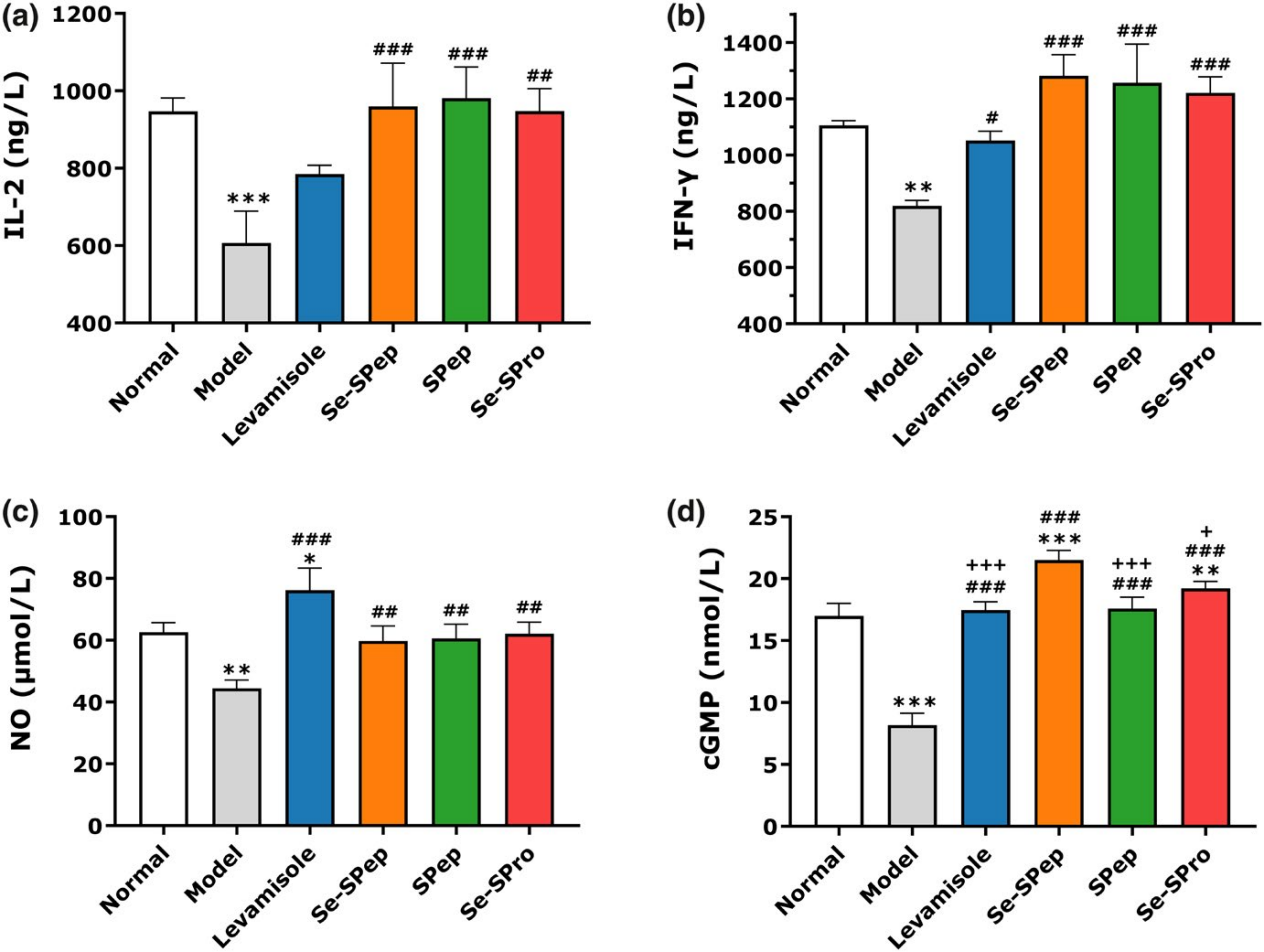
Effect of Se‐SPep on the IL‐2 (a), IFN‐γ (b), NO (c), and cyclic guanosine monophosphate (d) levels in the spleens of cyclophosphamide‐treated mice (*n* = 10). Compared with the Normal group: ∗*p* < .05, ∗∗*p* < .01, and ∗∗∗*p* < .001; compared with the Model group: ^#^ *p* < .05, ^##^ *p* < .01, and ^###^ *p* < .001; compared with the Se‐SPep group: ^+^ *p* < .05 and ^+++^ *p* < .001

The levels of IL‐2, IFN‐γ, NO, and cGMP in the spleen were determined to further demonstrate the immunomodulatory activity of Se‐SPep. Figure 5 shows that the IL‐2, IFN‐γ, NO, and cGMP levels in the spleens of the Model group were significantly reduced compared with the Normal group, indicating that CTX could significantly inhibit the production of cytokines, NO, and cGMP. The IFN‐γ, NO, and cGMP concentrations in the Levamisole group increased markedly compared to the Model group.

Furthermore, treatment with Se‐SPep, SPep, and Se‐SPro substantially promoted the production of IL‐2, IFN‐γ, NO, and cGMP in the spleen. Se‐SPep, in particular, significantly improved the secretion of cGMP compared with the SPep and Se‐SPro groups. These results indicated that Se‐SPep could improve immune responses by increasing the secretion of cytokines, NO, and cGMP in spleens of CTX‐induced immunosuppressed mice.

### 3.7 The effect of Se‐SPep on gene expression

**FIGURE 6:**
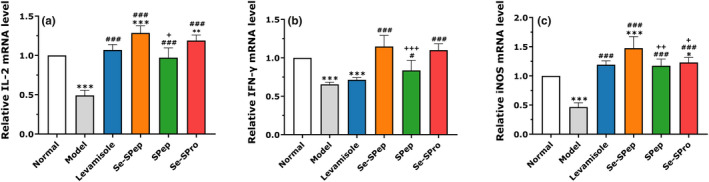
Effect of Se‐SPep on the mRNA expression levels of IL‐2 (a), IFN‐γ (b), and inducible nitric oxide synthase (c) in the spleens of the cyclophosphamide‐treated mice (*n* = 10). Compared with the Normal group: ∗*p* < .05, ∗∗*p* < .01, and ∗∗∗*p* < .001; compared with the Model group: ^#^ *p* < .05 and ^###^ *p* < .001; compared with the Se‐SPep group: ^+^ *p* < .05, ^++^ *p* < .01, and ^+++^ *p* < .001

To further confirm the immune enhancement of Se‐SPep in the spleen, qRT‐PCR was performed to examine the induction of the transcriptional upregulation of IL‐2, IFN‐γ, and iNOS. As shown in Figure 6, the mRNA expression of IL‐2, IFN‐γ, and iNOS in the spleens of the Model group was significantly lower than in the Normal group. Compared with the Model group, the mRNA expression of IL‐2 and iNOS was substantially higher in the spleens of the Levamisole group. Furthermore, Se‐SPep, SPep, and Se‐SPro treatment remarkably increased the related gene expression compared to the Model group, which was consistent with the IL‐2, IFN‐γ, and NO levels. Compared with the SPep group, the mRNA expression of IL‐2 and IFN‐γ increased significantly in the Se‐SPep group. The Se‐SPep group showed substantially elevated mRNA levels of iNOS in comparison with the SPep and Se‐SPro groups. Therefore, Se‐SPep could promote the secretion of IL‐2, IFN‐γ, and NO by enhancing the gene expression of IL‐2, IFN‐γ, and iNOS.

## 4 DISCUSSION

The immune function of the body is performed by a complete immune system consisting of immune organs (bone marrow, spleen, and thymus, etc.), immune cells (lymphocytes, phagocytic cells, and neutrophils, etc.), and immune molecules (immunoglobulins, interferon, interleukins, and tumor necrosis factors, etc.) (Wang et al., 2016). The immune system is responsible for maintaining a homeostatic immune balance by controlling and regulating the autoimmune response, which is essential for human health (Zhang, Gong, et al., 2020). Disorders of the immune system can result in autoimmune diseases, inflammatory diseases, and even cancer (Gao et al., 2019). Immunomodulators can restore the immune responses in the body to normal levels (Mohamed et al., 2017), alleviating and treating various diseases caused by low immune functionality. It is difficult to obtain an accurate assessment regarding the effect of immunomodulators or nutritional supplements on healthy humans and animals (Huang et al., 2007). Therefore, immunosuppressive agent‐induced immunosuppressed mice models have been established to evaluate the immunomodulatory effect of immunomodulators or nutritional supplements in vivo.

Cyclophosphamide is a cytotoxic chemotherapeutic drug, playing a crucial role in tumor treatment. However, CTX treatment can cause many adverse reactions, including immunosuppression and myelosuppression (Wang et al., 2011). The administration of a specific CTX dose over a short period has been shown to significantly reduce the immune function of mice (Zhu et al., 2018). Therefore, an immunosuppressed mouse model was established in this study via the intraperitoneal injection of 80 mg/kg of CTX for two consecutive days to evaluate the immunomodulatory effect of Se‐SPep from three perspectives: nonspecific immunity, cellular immunity, and humoral immunity. After 2 days of CTX administration, the bodyweight and immune organ indexes were significantly reduced. The WBC, RBC, HGB, and HCT, and the secretion of the IL‐2, IFN‐γ, NO, and cGMP in the spleen, as well as the mRNA expression of IL‐2, IFN‐γ, and iNOS in the spleen, were significantly lower than those in the Normal group. In addition, the serum immunoglobulin levels and plasma protein values decreased following CTX administration, indicating that the CTX‐induced immunosuppressed mouse model was established successfully.

The functions of the spleen and thymus are closely related to cellular and humoral immunity (Zhu et al., 2016). The spleen provides a place for the immune cells to settle, where they receive antigen stimulation to produce an immune response (Sun et al., 2019). The thymus is the site of T‐lymphocyte differentiation and maturation and is involved in regulating peripheral T‐cell maturation (Li et al., 2017). The increased weight of the spleen and thymus represents the proliferation of lymphocytes in the organs, while decreased immune function may be due to organ atrophy (Zhao et al., 2018). CTX inhibits the differentiation of lymphocytes, reducing their number in the immune organs, which results in a decrease in the weight of the spleen, thymus, and body. Immuno potentiators or nutritional supplements can increase the weight of these organs. Therefore, the effect of nutritional supplements on the spleen and thymus indexes can be used as preliminary indicators to examine the immunopharmacological mechanisms in animal models. This study showed that preventative Se‐SPep and Se‐SPro treatment alleviated the atrophy of the thymus and weight loss observed in immunosuppressed mice. Moreover, the thymus indexes of Levamisole, Se‐SPep, and Se‐SPro groups did not display a significant decrease compared with those of the Normal group. This indicated that Se‐SPep and Se‐SPro treatment improved the atrophy of the thymus caused by CTX while displaying an apparent immunity‐enhancing effect on the immune organs. Consistent with various previous studies, Se can increase the spleen and thymus indexes in CTX‐induced immunosuppression mouse models. The higher index values in CTX‐treated mice treated with Se‐enriched *Grifola frondosa* (*G. frondosa*) polysaccharides (Se‐GFP‐22) indicated that Se‐GFP‐22 was able to protect against the CTX‐induced atrophy of the immune organs (Li et al., 2018). Yuan et al. (2015) found that Se‐enriched green tea polysaccharides significantly improved the thymus and spleen indexes of mice compared with ordinary green tea polysaccharides.

White blood cells plays an important role in the defense system of the body, swallow foreign matter and generate antibody (Liu et al., 2019). Changes in the number of WBC helps to assess immunocompromised diseases and acute infectious diseases (Wu et al., 2019). However, the lifetime of WBC is short, and it requires bone marrow stem cells for continuous differentiation (Arslan et al., 2014). Bone marrow is a hematopoietic organ and is central to human and mammal immunity. Furthermore, bone marrow suppression is one of the most obvious side effects of chemotherapy drugs (Han et al., 2019). Hematopoietic depression involves all blood elements: leukocytes, platelets, and RBCs (Kaya et al., 2019). In this study, the hematological values in CTX‐treated mice showed myelosuppressive changes, specifically in WBC, RBC, HGB, and HCT. However, the WBC, RBC, HGB, and HCT of mice treated with Se‐SPep and Se‐SPro were significantly increased compared to the Model group. The results indicated that Se‐SPep and Se‐SPro have an immunoprotective effect on CTX‐treated mice by improving myeloid hematopoiesis on the CTX‐induced myelosuppression.

The detection of serum protein is vital during the identification of clinical, biochemical indicators. ALB is the most important protein in human blood, accounting for about 50% of the TP, which maintains nutrition and osmotic pressure in the body (Soeters et al., 2019). In this study, the preventive treatment with Se‐SPep, SPep, and Se‐SPro significantly enhanced the TP and ALB levels in the serum of the mice, compared with the Model group. The results indicated that nutritional supplementation with protein could maintain a higher plasma protein level in mice, which is conducive to the synthesis of immune proteins, exerting a strong immune preventive effect. Humoral immunity, a critical vertebrate immunity response, is mediated via B lymphocytes. Immunoglobulins are proteins produced after the proliferation and differentiation of B cells in response to a specific antigen (Gao et al., 2019). Immunoglobulins play a crucial role in humoral immunity and can recognize and neutralize foreign substances (Meng et al., 2017). Secreted IgG, IgM, and IgA are the major antibody components in serum and are essential effector molecules in humoral immune responses (Wang et al., 2011). IgM represents the primary antibody produced during the early stage of the initial humoral immune response and exhibits a strong anti‐infection effect (Ehrenstein & Notley, 2010). IgG, the most abundant antibody in sera, is critically involved in the phagocytosis mechanism of monocytes (Nimmerjahn & Ravetch, 2010). Sera IgA triggers the function of an effector protein with the potential to eliminate exogenous microorganisms (Woof & Kerr, 2006). In this study, the IgM, IgG, and IgA levels of the Se‐SPep group were significantly enhanced compared to the Model group. Similar results were observed in several previous studies regarding sodium selenite (Xu et al., 2015), Se‐enriched alfalfa hay (Wallace et al., 2017), and Se yeast (Mansour et al., 2017) in CTX‐induced immunosuppressed mice. Therefore, Se‐SPep can enhance the humoral immune response by increasing immunoglobulin production while maintaining homeostasis by reversing the immune disorder. Moreover, Se‐SPep provides a sufficient protein source for producing immunoglobulins.

Cytokines act via receptors and are especially important in the immune system, including the inflammatory response, cell proliferation, and apoptosis (Hirja et al., 2019). They modulate the balance between humoral and cell‐based immune responses and regulate the maturation, growth, and responsiveness of specific cell populations (Cabral et al., 2015). The induction of cytokine synthesis is one of the methods to evaluate the augmentation activity of innate immunity (Zhang, Gong, et al., 2020). Therefore, to determine the level of the body's immune function, the key is to detect the secretion level of cytokines (Niu et al., 2020). IFN‐γ and IL‐2 are primary immunity‐related cytokines. IFN‐γ, mainly produced by NK cells and Th1, is considered part of innate immunity and antigen‐specific immunity (Ding et al., 2019). IFN‐γ can mediate cellular immune function and promote the differentiation and proliferation of Th1 cells (Schroder et al., 2004). IL‐2 is one of the most important immune factors that induce the differentiation of lymphocyte cells to participate in the immunomodulatory effect (Gaffen & Liu, 2004). IL‐2 promotes T‐cell growth, enhances NK‐cell lytic activity, and induces the differentiation of Treg cells (Park & Lee, 2018). Figure 5 shows that the administration of Se‐SPep, SPep, and Se‐SPro significantly increased the spleen concentrations of INF‐γ and IL‐2 in CTX‐treated mice. Luan et al. confirmed that sufficient Se supplementation reduced the synthesis of pro‐inflammatory cytokines, promoting the secretion of IFN‐γ and IL‐2 by lymphocytes (Luan et al., 2016). Egusa and Otani (2009) confirmed that soybean peptides could significantly promote the secretion of INF‐γ and IL‐2 content in mice splenocytes. As shown in Figure 6, the mRNA expression of INF‐γ and IL‐2 in the spleen of the Se‐SPep, SPep, and Se‐SPro groups was significantly increased, compared with the Model group. YAO et al. indicated that Se deficiency could reduce the number of T cells and the expression of IL‐2 (Yao et al., 2016). Hoffmann et al. fed mice with a high‐Se diet and found that the expression of IL‐2 mRNA and the IFN‐γ concentration in the high‐Se group increased substantially. Furthermore, the Th1/Th2 ratio shifted to the Th1 phenotype (Hoffmann et al., 2010). In this study, supplementation with Se‐SPep improved the reduction of IFN‐γ and IL‐2 caused by CTX‐induced immunosuppression by upregulating the mRNA expressions of INF‐γ and IL‐2. Therefore, when the immunostimulatory Se‐SPep activities are executed, IFN‐γ and IL‐2 may be induced, acting as immune regulators to modulate innate and adaptive immune responses. Se‐SPep may have the potential to enhance the immune system via Th1 phenotype potentiation.

Nitric oxide, synthesized from l‐arginine by iNOS, is an important signaling medium in organisms and is involved in many biological functions, such as vasodilatation, neurotransmission, immune response, and platelet aggregation (Lee et al., 2015). INOS is the primary enzyme responsible for NO production (Jiang et al., 2011). The augmentation of iNOS activity can enhance NO production in activated macrophages (Kong et al., 2014). After NO is produced, it rapidly diffuses to adjacent target cells via biofilm and bodily fluids, increasing cGMP synthesis by activating guanosine cyclase (GC). It is widely accepted that cAMP and cGMP are second messengers, playing crucial cellular signal transduction roles (Zhang et al., 2014). In general, cAMP exerts a suppressive effect on the functions of inflammatory and immunocompetent cells (Horrigan et al., 2006). CGMP can promote the proliferation of lymphocytes while performing positive immunoregulatory functions (Dai et al., 2009). In this study, the secretion of NO and the expression of iNOS mRNA in the Se‐SPep, SPep, and Se‐Spro groups were significantly higher than in the Model group. Moreover, cGMP production in the Se‐SPep, SPep, and Se‐Spro groups was significantly higher than the Model group. Li et al. (2018) confirmed that the Se‐enriched *G. frondosa* could significantly increase iNOS mRNA expression in the spleens of immunosuppressed mice. NO may be induced by upregulating the expression of iNOS. The cAMP and cGMP cascades may be inhibited and enhanced, respectively, subsequently affecting the expression of downstream immunosignal molecules. Therefore, Se‐SPep can promote the gene expression of iNOS, implying that Se‐SPep may induce NO production by up‐regulating the expression of iNOS when exerting its immunomodulatory functions. Furthermore, enhancing the cGMP cascade and subsequently affecting the expression of downstream immunosignal molecules may be one of the immunomodulatory mechanisms of Se‐SPep.

## 5 CONCLUSIONS

In summary, this study provides in vivo evidence that Se‐SPep has an immunomodulatory effect on CTX‐induced immunosuppression in mice. Se‐SPep significantly increases bodyweight while improving the spleen and thymus indexes. Moreover, peripheral WBC, RBC, HGB, and HCT showed that Se‐SPep treatment inhibits CTX‐induced immunosuppression. The serum TP and ALB levels are restored compared to those in immunosuppressed mice. The secretion of IgM, IgG, and IgA are adjusted following Se‐SPep treatment, significantly increasing the production of IL‐2, IFN‐γ, NO, and cGMP in the spleen, as well as the mRNA expression of IL‐2, IFN‐γ, and iNOS. This suggests that Se‐SPep can enhance the specific and nonspecific immunity of the host, including cellular and humoral immune systems. Furthermore, compared to SPep and Se‐SPro, Se‐SPep exhibits more effective immunomodulatory activity. These observations indicate that Se‐SPep is a potent immunopotentiating and immunomodulatory agent.

## CONFLICT OF INTEREST

The authors declare that they do not have any conflict of interest.

## AUTHOR CONTRIBUTIONS

**Jian Zhang:** Data curation (equal); Investigation (equal); Methodology (equal); Writing‐original draft (equal). **Siwei Gao:** Data curation (equal); Methodology (equal); Writing‐original draft (supporting). **He Li:** Conceptualization (equal); Data curation (equal); Writing‐review & editing (equal). **Mengdi Cao:** Visualization (equal). **Wenhui Li:** Writing‐review & editing (equal). **Xinqi Liu:** Conceptualization (equal); Writing‐review & editing (equal).

## ETHICAL APPROVAL

This study was approved by the Institutional Animal Care and Use Committee at the Pony Testing International Group Co., Ltd. (PONY‐2021‐FL‐02).

## Data Availability

The data that support the findings of this study are available from the corresponding author upon reasonable request.
